# Spectral Domain Sparse Representation for DOA Estimation of Signals with Large Dynamic Range

**DOI:** 10.3390/s21155164

**Published:** 2021-07-30

**Authors:** Jacob Compaleo, Inder J. Gupta

**Affiliations:** ElectroScience Laboratory, The Ohio State University, Columbus, OH 43212, USA; gupta.11@osu.edu

**Keywords:** direction-of-arrival (DOA) estimation, sparse representation, beamforming, apodization, window function

## Abstract

Recently, we proposed a Spectral Domain Sparse Representation (SDSR) approach for the direction-of-arrival estimation of signals incident to an antenna array. In the approach, sparse representation is applied to the conventional Bartlett spectra obtained from snapshots of the signals received by the antenna array to increase the direction-of-arrival (DOA) estimation resolution and accuracy. The conventional Bartlett spectra has limited dynamic range, meaning that one may not be able to identify the presence of weak signals in the presence of strong signals. This is because, in the conventional Bartlett spectra, uniform weighting (window) is applied to signals received by various antenna elements. Apodization can be used in the generation of Bartlett spectra to increase the dynamic range of the spectra. In Apodization, more than one window function is used to generate different portions of the spectra. In this paper, we extend the SDSR approach to include Bartlett spectra obtained with Apodization and to evaluate the performance of the extended SDSR approach. We compare its performance with a two-step SDSR approach and with an approach where Bartlett spectra is obtained using a low sidelobe window function. We show that an Apodization Bartlett-based SDSR approach leads to better performance with just single-step processing.

## 1. Introduction

The origins of direction-of-arrival (DOA) estimation research can be traced back to World War II. Bartlett’s method [1] is one of the earliest DOA estimation techniques that is still in use today. Bartlett’s method is successful in estimating DOA in single-emitter scenarios. In multiple-emitter scenarios, Bartlett’s method becomes biased as the angular spacing between emitters becomes small. In small angular separation scenarios, a high-resolution DOA estimation technique is desired for unbiased estimates.

The multiple signal classification (MUSIC) [2] algorithm is a widely used high-resolution DOA estimation algorithm. The MUSIC algorithm provides a method to estimate the DOAs of multiple emitters in a single scene at a high resolution and with a large success rate. The MUSIC algorithm has many advantages compared to classic DOA estimation techniques such as Bartlett’s method, though it has some drawbacks of its own. In scenarios with a low number of snapshots being utilized as well as in cases with low angular separation between emitters, MUSIC’s performance begins to suffer and the algorithm fails to resolve signals [3].

Sparse representation and its minimization capabilities have also made it a popular tool for DOA estimation. Many examples of sparse-representation applications for DOA estimation can be found in the literature. Oftentimes, sparse representation is applied in the data domain to the covariance matrix [4,5,6,7,8,9,10,11,12,13,14,15]. Other recent popular uses of sparse representation for improved DOA estimation include the use of Bayesian Learning [16,17,18,19,20,21,22] or co-prime and nested arrays [23,24,25,26,27,28,29,30]. Recent work focusing on the application of interpolation to decrease the off-grid effects of sparse representation has also been presented [31,32,33]. While these referenced papers present differing methods for applying sparse representation, they all show the clear advantage that the use of sparse representation provides for DOA estimation performance.

In [34], we presented a sparse-representation approach for DOA estimation that utilizes Bartlett’s method as a forward model and starting point. We call this approach the Spectral Domain Sparse Representation (SDSR) method. The SDSR method is based on the assumption that the Bartlett spectra computed using the received signals is a superposition of the Bartlett spectra of individual RF emitters. The superposition assumption allows us to create a dictionary matrix of Bartlett spectra. We can then compare a Bartlett spectra of interest with this dictionary matrix using sparse representation to achieve DOA estimation performances with increased resolution and accuracy. While there are other approaches that combine beamforming and sparse representation such as [35], the SDSR method differs from others as we focus solely on the major lobes of the Bartlett spectra and use those as a starting point for sparse representation. This helps us to mitigate off-grid errors.

In [34], we focused solely on scenarios where all signals had equal strength. We showed that the SDSR method outperforms the MUSIC algorithm when angular separation between signals is small, SNR is low, and a small number of snapshots are used for DOA estimation. When there is a large dynamic range amongst signals in a given environment, the performances of many DOA algorithms suffer. Often, the weaker signals are buried in noise and cannot be accurately estimated. This issue is apparent with Bartlett’s method, where the major lobe of the strong signal oftentimes encompasses that of the weaker signal. As many real-world scenarios involve signals of differing strengths, DOA algorithms robust to signals with a large dynamic range are of interest.

Instances of DOA algorithms robust to signals with a large dynamic range can be found throughout the literature [36,37,38]. In [36], the extended noise subspace of the MUSIC algorithm is utilized to estimate the DOA of weak signals in the presence of strong interferers. Reference [37] focused on signals with large power differences that are also closely spaced. The eigen spatial spectrum was utilized in this proposed approach. In [38], an iterative approach that uses digital beamforming was proposed to first estimate the DOA of the strongest signal and then the weaker signals follow after.

In our previous work, we used the conventional Bartlett’s method, where equal weighting is applied to the signal received at each antenna element. Therefore, it can be said that we applied a uniform window function. This uniform window function is insufficient at estimating signals with a large dynamic range as the weaker signal is often buried in the sidelobes of the stronger signal. While there are other window functions that could be applied to the Bartlett spectra (Chebyshev, Hamming, Kaiser, etc.), these window functions effectively reduce the aperture size and can lead to decreased performance when compared with the uniform window function. In this work, we propose the use of a mixed window approach that should maintain the performance of the uniform window approach while also possessing the capability to resolve signals with a large dynamic range. This mixed window approach is also commonly referred to as Apodization [39]. In this work, we present a method for extending the SDSR method for scenarios with signals with large dynamic ranges by applying Apodization to Bartlett’s method. While multiple window functions can be used in Apodization, we use just two window functions in this work: the uniform window and Chebyshev window. We show that utilizing Apodization with the SDSR method is straightforward and provides promising results. Using Monte Carlo simulations, we demonstrate that the extended SDSR method that utilizes Apodization is both efficient and accurate at estimating DOA for signals with large dynamic range. We compare the performance of the Apodization extended SDSR method with the single-windowed SDSR approach, where a low-sidelobe Chebyshev window is applied to Bartlett’s method. We also compare the Apodization approach with a two-step SDSR modification that requires running the SDSR method twice and subtracting strong signal information to obtain information about weak signals. We show that the Apodization SDSR modification has a better performance than the windowed SDSR modification. Additionally, the Apodization SDSR modification has less failed trials than the two-step SDSR method when compared using Monte Carlo simulations. We show that, in two-, three-, and four-signal scenarios with large dynamic ranges, the Apodization SDSR modification provides the best overall performance of the three modifications in terms of having a small number of failures and maintaining a low Root Mean Square Error (RMSE).

The remainder of this paper is organized in the following way. Section 2 discusses the basic signal model setup for DOA estimation. Section 3 is a review of the SDSR method. Section 4 presents the three proposed modifications to the SDSR method that make it robust to signals with large dynamic ranges. Section 5 shows the Monte Carlo simulation results from comparing the three proposed modifications and the Cramer–Rao bound for two-, three-, and four-signal scenarios. Lastly, in Section 6, we discuss our results and provide a conclusion.

## 2. Signal Model

Let *K* overlapping narrowband signals be received by an *N* element antenna array. For this work, overlapping means that the signals are received at the same frequency and time. The received signal at the *i*th element can be represented as [40]

(1) $$y_{i}\left(t\right)=\left(\sum_{k=1}^{K}d_{i}\left(\phi_{k}\right)s_{k}\left(t\right)\right)+\nu_{i}\left(t\right)$$

where $s_{k}\left(t\right)$ is the *k*th signal received by an isotropic antenna located at the coordinate origin (the phase reference point of the antenna array), $d_{i}\left(\phi_{k}\right)$ is the gain and phase shift of the *i*th element of the antenna in the emitter direction $\phi_{k}$, and $\nu_{i}\left(t\right)$ is the thermal noise. Note that $d_{i}\left(\phi_{k}\right)$ includes the phase shift due to the element being not at the coordinate origin. We assume that the noise is uncorrelated with the incident signals and between the various antenna elements. Further noise is assumed to be complex circular Gaussian with unity variance. For all *N* elements, (1) can be written in vector form as

(2) $$y\left(t\right)=\left(\sum_{k=1}^{K}d\left(\phi_{k}\right)s_{k}\left(t\right)\right)+\nu \left(t\right)$$

where y(t) is the received signal vector of length *N*, $d(\phi_{k})$ is referred to as the antenna array manifold vector of length *N*, and ν(t) is the noise vector of length *N*. The equation can be represented in matrix form to remove the summation as

(3) y(t)=Ds(t)+ν(t)

where

$$D=[d\left(\phi_{1}\right),\ldots ,d\left(\phi_{K}\right)]$$

$$s\left(t\right)={\left[s_{1}\left(t\right),\ldots ,s_{K}\left(t\right)\right]}^{T}$$

where D is a matrix of size *N* by *K* and s(t) is a vector of length *K*. The received signals are downconverted and digitized using analog-to-digital converters (ADC). Let the signal be digitized with a sampling period of *T* seconds. The *l*th sample after digitization can be represented as

(4) y[l]=Ds[l]+ν[l]

where (lT) is written concisely as [l]. y[l] is referred to as the snapshot vector. Let *P* snapshots be used to estimate the DOA. The snapshot vectors can be represented in matrix form as

(5) Y=[y[1],…,y[P]]

where Y is now referred to as the snapshot matrix.

The popular DOA estimation technique, Bartlett’s method, uses the antenna array manifold and snapshot matrix to estimate the signal direction. The conventional Bartlett spectrum can be computed as

(6) $$b\left(\phi \right)=\frac{d{\left(\phi \right)}^{H}\hat{R}d\left(\phi \right)}{d{\left(\phi \right)}^{H}d\left(\phi \right)}$$

where d(ϕ) is the antenna array manifold in the direction ϕ and $\hat{R}$ is the sample covariance matrix. The sample covariance matrix is represented as

(7) $$\hat{R}=\frac{1}{P}YY^{H}$$

where *H* is the Hermitian transpose. In practical situations such as scenarios with a low number of snapshots available, robust estimators can be used in place of the sample covariance matrix to improve performance [41,42,43,44]. The peaks in the Bartlett spectrum correspond to the direction of the incident signals.

## 3. Review of SDSR Method

In [34], we presented a high-resolution sparse representation-based DOA estimation method, the SDSR method, that uses the conventional Bartlett spectra as a forward model and starting point. Initially, the observed Bartlett spectra, b, is generated. After identifying the major lobes of b, the angular regions within the major lobes are populated with many closely spaced emitters with signal strength unity. An individual Bartlett spectra is calculated for each of these emitters. For a unit power emitter located along direction $\phi_{m}$, the Bartlett spectra in direction ϕ is given by

(8) $$a_{m}\left(\phi \right)=\frac{|d^{H}\left(\phi \right)d\left(\phi_{m}\right){|}^{2}}{\left(d^{H}\left(\phi \right)d\left(\phi \right)\right)}$$

m=1,2,…,M

where *M* is the total number of emitters distributed in the angular regions of interest. These *M* Bartlett spectra form the dictionary of interest. In the SDSR method, one selects a few elements of this dictionary to represent the observed Bartlett spectra, b. One can then find the unknown signal strengths by solving the following well-known optimization problem [45]

(9) $$min{\left\|x(\beta )\right\|}_{1}$$

such that

(10) $${\left\|Ax(\beta )-b\right\|}_{2}^{2}<\beta {\left\|b\right\|}_{2}^{2}$$

where ${\left\|.\right\|}_{1}$ is the L1-norm and ${\left\|.\right\|}_{2}^{2}$ is the square of the Euclidean norm, also referred to as the L2-norm. A is a matrix of *M* columns, with each column representing the Bartlett spectra of an individual emitter of signal strength unity, as computed in (8). x(β) of length *M* represents the signal strength of the individual emitters that leads to the desired match between the observed spectra and combined spectra of the individual emitters and β is the sparse representation regularization parameter. Our method for selecting β is reported in [34]. The additional constraint that all entries of vector x(β) must be positive was also included. The MATLAB optimization package SeDuMi [46] was used to solve the constraint minimization problem.

**Figure 1:**
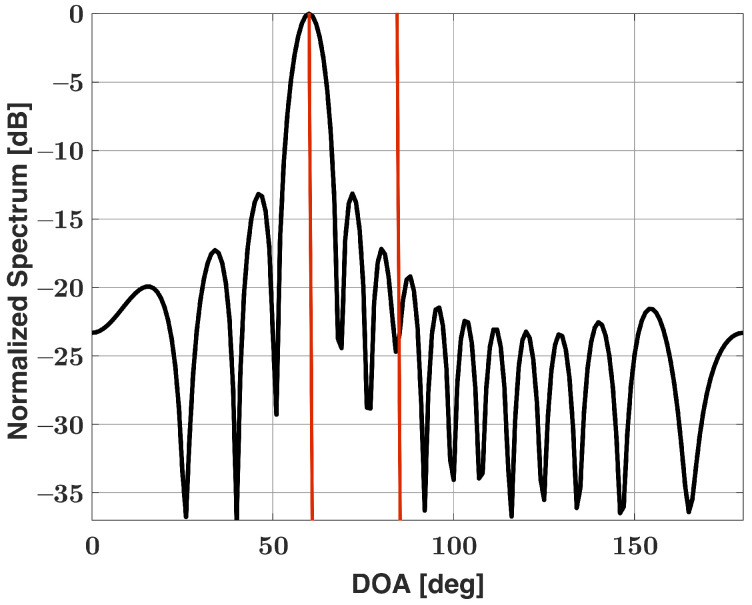
Bartlett spectra for a uniform linear array of 15 elements in the presence of two signals incident at 60° (25 dB SNR) and 85° (0 dB SNR).

In [34], we showed that the SDSR is an unbiased and efficient estimator for same-strength signals in a plethora of scenarios. As mentioned, the proposed SDSR method utilizes the conventional Bartlett spectra as a forward model and starting point. When signals of greatly differing strengths are introduced in the same scene, the conventional Bartlett’s method often fails to detect the weaker signal. The weaker signal becomes buried in the sidelobes of the stronger signal. Figure 1 shows the Bartlett spectra for a linear uniform antenna array of fifteen isotropic elements spaced half a wavelength apart in the presence of two signals incident on the antenna array. One hundred snapshots are used to obtain the Bartlett spectra. The first signal is incident at 60° and has a signal-to-noise ratio (SNR) of 25 dB. The second signal is incident at 85° and has an SNR of 0 dB. The red lines in the figure correspond to the true directions of the signals. Note that, despite there being two signals with large angular separation incident on the antenna array, only one major lobe is apparent in the figure. The weaker signal that is incident at 85° is buried in the sidelobes of the stronger signal and is not noticeable. The conventional Bartlett’s method fails to resolve the two signals here and is no longer feasible to be used as a starting point for our method in its current form. In the following section, we present three potential modifications to the SDSR algorithm to estimate the DOA of signals with large dynamic range.

## 4. Modifications to SDSR Method

### 4.1. Two-Step Method

**Figure 2:**
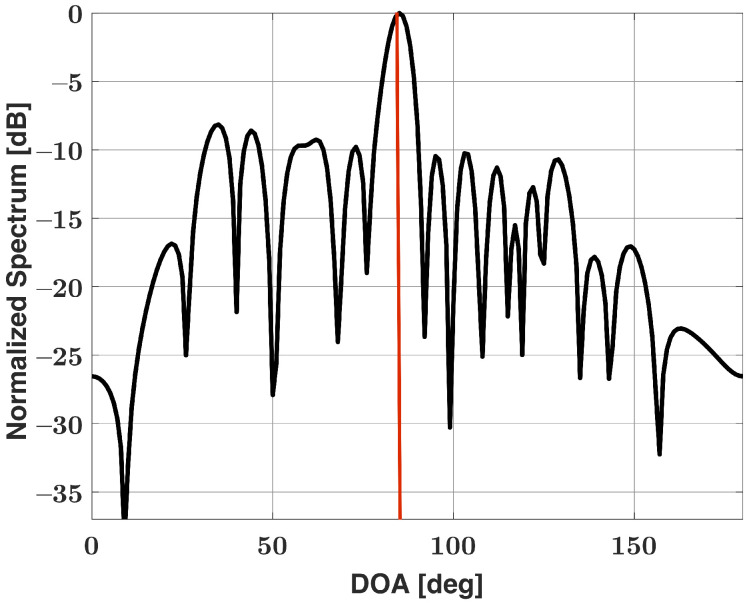
Residual Bartlett spectra for a uniform linear array of 15 elements in the presence of two signals incident at 60° (25 dB SNR) and 85° (0 dB SNR): the estimated signal incident at 60° with an SNR of 25 dB has been subtracted.

As we previously stated, our method works under the assumption that the observed Bartlett spectra is a superposition of the spectra of individual emitters. We can use this assumption once again to modify the SDSR method for emitter scenarios that involve signals with large dynamic ranges. Using the Bartlett spectra shown in Figure 1 as a starting point for our algorithm, we focus in around the major lobe regions and obtain DOA estimates as well as the strength of the strong signals that are contained in the major lobes. In general, there may be multiple strong signals present in a scene and each corresponding major lobe would be of interest for our algorithm. For this example, our algorithm estimates a 25 dB signal incident at 60°. Using the superposition assumption, we can subtract the individual Bartlett spectra associated with strong signals from the observed Bartlett spectra b (shown in Figure 1 for this example) to check for weak signals buried in the sidelobes of the strong signals. Mathematically, this can be expressed as

(11) $$\tilde{b}=b-\sum_{k=1}^{K}a\left(\phi_{k}\right)x\left(\phi_{k}\right)$$

where b is the observed Bartlett spectra, *K* is the number of strong signals present in the scene, and $\tilde{b}$ is the residual Bartlett spectra. $a\left(\phi_{k}\right)x\left(\phi_{k}\right)$ represents the individual response for the *k*th strong signal incident in direction $\phi_{k}$ (obtained using the SDSR method). Note that (11) differs from the popular Matching pursuit algorithm [47] because we are not limited to subtracting just a single strong signal to obtain the residual and can feasibly subtract multiple strong signals at once if necessary, unlike the Matching pursuit algorithm. Figure 2 shows the residual Bartlett spectra $\tilde{b}$ after the Bartlett spectra of the 25 dB SNR signal incident at 60° has been subtracted. From Figure 2, it is now clear that there is a second and much weaker signal present in the scene. We now can use this residual spectra as the starting point for the SDSR algorithm and can obtain a DOA estimate as well as the strength of the weaker signals. This is a two-step approach that requires running our algorithm twice. For this example, the Bartlett spectra shown in Figure 1 first operates as the b in (10) for step one, and then, the residual spectra $\tilde{b}$ shown in Figure 2 is utilized in place of b in (10) for step two. As the first step has already been expressed mathematically in Section 3, let the use of our algorithm in step two be expressed as

(12) $$min{\left\|\tilde{x}\left(\beta \right)\right\|}_{1}$$

such that

(13) $${\left\|\tilde{A}\tilde{x}\left(\beta \right)-\tilde{b}\right\|}_{2}^{2}<\beta {\left\|\tilde{b}\right\|}_{2}^{2}$$

where $\tilde{A}$ is a dictionary matrix focused on the major lobes of $\tilde{b}$ and where $\tilde{x}\left(\beta \right)$ contains the DOAs and signal strengths of the weaker signals. With the proposed two-step modification, our algorithm should be able to accurately estimate DOA despite the large dynamic range between signals.

### 4.2. Windowed Bartlett Spectra

**Figure 3:**
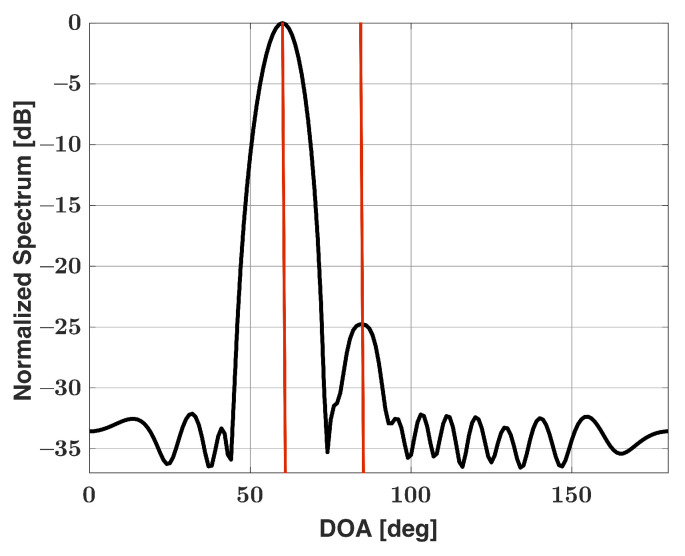
Bartlett spectra for a uniform linear array of 15 elements in the presence of two signals incident at 60° (25 dB SNR) and 85° (0 dB SNR), Chebyshev window applied.

Rather than perform two steps for our algorithm, one can use a low sidelobe window function to generate the Bartlett spectra. For conventional Bartlett’s method, we apply equal weighting to the signal received at each antenna element. Therefore, it can be said that we apply a uniform window function. As is widely known, uniform windows provide the best angular resolution of window functions, though at the cost of having large sidelobes compared with other window functions. A window function that greatly reduces sidelobes is more ideal for signals with a large dynamic range. Let us again work with the Bartlett spectra from Figure 1. Instead of uniform weighting, we apply a Chebyshev window with 35 dB sidelobe suppression to the received data. The new windowed Bartlett spectra is expressed as

(14) $$b^{w}\left(\phi \right)=\frac{{\left(Wd(\phi )\right)}^{H}\hat{R}\left(Wd(\phi )\right)}{(W{d(\phi ))}^{H}\left(Wd(\phi )\right)}$$

where W is a matrix of size N×N with window function values along the diagonal. As stated, the Chebyshev window was chosen for this work. Figure 3 shows the resultant Bartlett spectra after the Chebyshev window has been applied. Note that two major lobes are now clearly present, albeit one is much weaker than the other. For our algorithm, we apply the same Chebyshev window to each column of dictionary matrix A such as

(15) $$a_{m}^{w}\left(\phi \right)=\frac{{|\left(Wd\left(\phi \right)\right)}^{H}\left(Wd\left(\phi_{m}\right)\right){|}^{2}}{\left({\left(Wd\left(\phi \right)\right)}^{H}\left(Wd\left(\phi \right)\right)\right)}$$

m=1,2,…,M

and let the new windowed dictionary matrix be referred to as $A^{w}$. The windowed sparse representation optimization problem can now be expressed as

(16) $$min{x^{w}\left(\beta \right)}_{1}$$

such that

(17) $${A^{w}x^{w}\left(\beta \right)-b^{w}}_{2}^{2}<\beta {b^{w}}_{2}^{2}$$

where $x^{w}\left(\beta \right)$ contains the DOAs and signal strengths of the signals. With the Chebyshev window applied, it is now possible to complete our algorithm in one step. Additionally, we should be able to obtain an accurate DOA estimate for signals with a large dynamic range.

### 4.3. Mixed Windowed Bartlett Spectra

**Figure 4:**
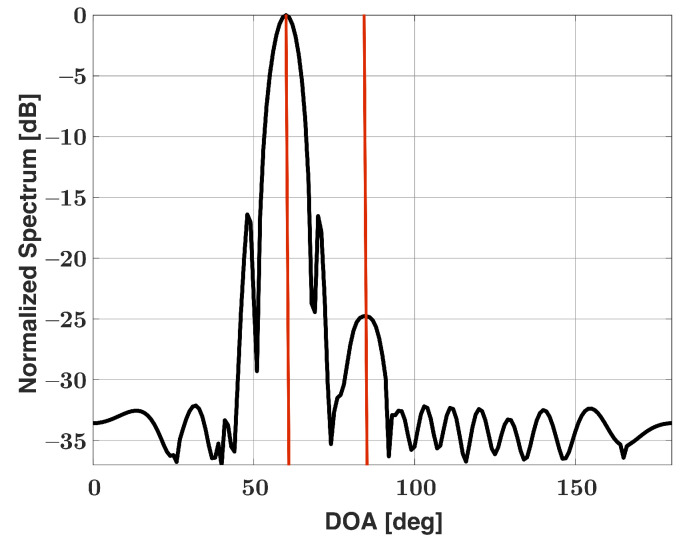
Bartlett spectra versus DOA for a uniform linear array of 15 elements in the presence of two signals incident at 60° (25 dB SNR) and 85° (0 dB SNR), dual apodization (Chebyshev and uniform windows applied).

Applying a nonuniform window function to the Bartlett spectra may improve the DOA estimation capabilities for signals with large dynamic ranges, though the modification has its own drawbacks. Nonuniform window functions are able to reduce sidelobe levels, albeit at the cost of potentially worse performance compared with uniform windows. For example, the Chebyshev window (as well as all other windows) has worse angular resolution when compared with the uniform window. Additionally, using a Chebyshev window effectively decreases aperture size, which can lead to worse performance. Therefore, the performance of our algorithm when using the Chebyshev window is expected to degrade in comparison with the performance achieved with the uniform window. Apodization, also known as the use of multiple window functions, presents a potential solution for maintaining angular resolution while also decreasing sidelobe levels [39]. Dual-apodization is utilized in this work, and the two selected window functions are the uniform and Chebyshev windows. Two Bartlett spectra are generated: one with uniform weights and one with Chebyshev weights. The two spectra are compared on a point-by-point basis at each angle ϕ, and the minimum value at each angle is stored. The concept related to this mixed windowing or apodization is that selecting the minimum value at each angle produces a new Bartlett spectra with a narrow major lobe similar to the conventional Bartlett spectra, while maintaining low sidelobe levels similar to the Chebyshev Bartlett spectra. Figure 4 shows the dual-apodized spectra for the scenario first shown in Figure 1. Two major lobes can be identified in the spectra, and it can also be noted that the major lobe is more narrow compared to Figure 3. Let the dual-apodized Bartlett spectra $b^{D}$ at angle ϕ be mathematically represented as

(18) $$b^{D}\left(\phi \right)=min\left\{b\left(\phi \right),b_{w}\left(\phi \right)\right\}$$

As with the previous modifications for our algorithm, we need to generate a new dictionary matrix that is applicable for the modification. Let us refer to the apodized dictionary matrix as $A^{D}$. From (18), we know which window function is applied at each angle ϕ to generate $b^{D}\left(\phi \right)$. Using this information, we generate $A^{D}$ with the appropriate window function applied to each column. This means that each direction ϕ in $b^{D}$ and $A^{D}$ should utilize the same window matrix W. The dual apodized sparse representation optimization problem can now be expressed as

(19) $$min{x^{D}\left(\beta \right)}_{1}$$

such that

(20) $${A^{D}x^{D}\left(\beta \right)-b^{D}}_{2}^{2}<\beta {b^{D}}_{2}^{2}$$

where $x^{D}\left(\beta \right)$ contains the DOAs and signal strengths of the signals. The mixed windowed SDSR modification is summarized in Algorithm 1. It is expected that the dual apodization spectra provides superior angular resolution and overall performance compared with the Chebyshev spectra.

**Algorithm 1** Mixed Windowed Bartlett Spectra SDSR **for**
i=1 to
length(ϕ) **do**
  $b^{D}\left(\phi \right)=min\left\{b\left(\phi \right),b_{w}\left(\phi \right)\right\}$
   Store which window function is the minimum at each angle ϕ. Let this window function be $w_{D}\left(\phi \right)$.
 
 **end for**
  Generate $A^{D}$ using the window function $w_{D}\left(\phi \right)$ for each assumed emitter location.
 
 $min{\left\|x^{D}\left(\beta \right)\right\|}_{1}$
  such that  ${\left\|A^{D}x^{D}\left(\beta \right)-b^{D}\right\|}_{2}^{2}<\beta {\left\|b^{D}\right\|}_{2}^{2}$


## 5. Results

We performed Monte Carlo simulations to evaluate the performance of the three modifications to the SDSR method for DOA estimation for signals with large dynamic ranges. For each signal scenario, 500 independent trials were carried out. The results of these trials were used to calculate the bias and RMSE in the estimated DOA. Bias is defined as

(21) $$Bias\left(\phi_{k}\right)={\bar{\phi }}_{k}-\phi_{k}$$

where $\phi_{k}$ is the true angle of signal *k* and where ${\bar{\phi }}_{k}$ is the mean estimate given by

(22) $${\bar{\phi }}_{k}=\frac{1}{J}\sum_{j=1}^{J}{\tilde{\phi }}_{k,j}$$

with ${\tilde{\phi }}_{k,j}$ being the angle estimate of the maximum for signal *k* and trial *j* and with *J* being the total number of trials (500 for this paper). RMSE is defined as

(23) $$RMSE\left(\phi_{k}\right)=\sqrt{\frac{1}{J}\sum_{j=1}^{J}{\left({\tilde{\phi }}_{k,j}-\phi_{k}\right)}^{2}}$$

**Figure 5:**
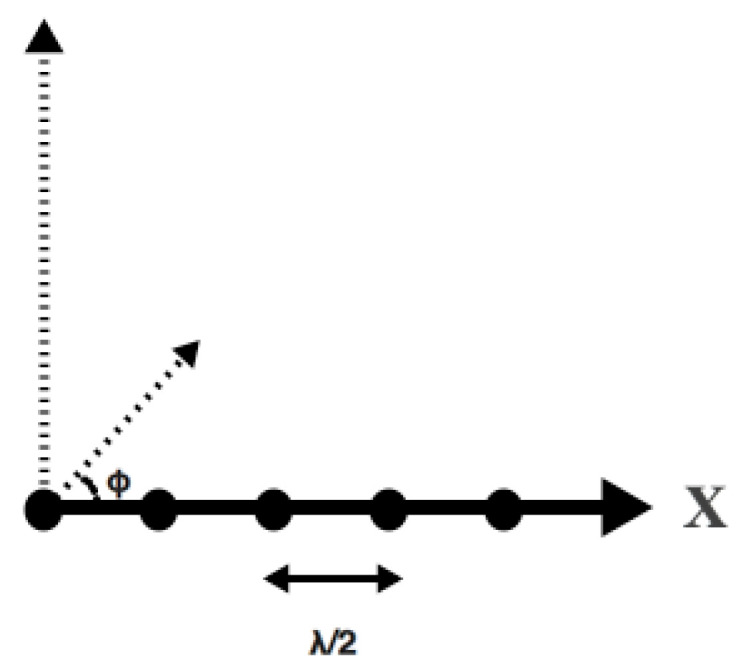
Uniform linear array of antenna elements placed along x-axis.

The DOA estimation performance for weaker signals in the scene is of interest in this work, and therefore, only bias and RMSE in the estimated DOA of the weaker signals are shown. The RMSE in the estimated DOA of the weaker signal is compared with the Cramer–Rao Bound (CRB) [48]. We used a uniform linear array of fifteen isotropic antenna elements in the simulations. The antenna elements were placed along the x-axis and had an interelement spacing of half a wavelength. All of the signals were incident in the plane of the paper. Along 60°, the null-to-null beamwidth of the antenna array is approximately 18 degrees. Thus, conventional methods are not able to resolve signals that have angular separations of less than 9 to 10 degrees. An angular separation less than this refers to the high-resolution region. The angle of arrival was measured with respect to the x-axis (see Figure 5). Therefore, 90° represents the broadside to the antenna array.

### 5.1. Two-Signal Scenario

Initially, two signals are incident to the antenna array, with fixed SNRs of 25 dB and 0 dB. Signal #1 is much stronger than signal #2; therefore, we can say that the dynamic range is large. Signal #1 is incident to the antenna array at 60°, and the direction of signal #2 varied from 65° to 85°, with a step size of 5°. One hundred snapshots are used in DOA estimation. The dual-apodization modification (uniform+Cheby), two-step modification (uniform), and the single window (Cheby) modification are compared. It is expected that, when angular separation is large, all three proposed modifications will have similar performance.

In some Monte Carlo trials, the weaker signal was not detected. We refer to these instances as failed trials. After removing these failed trials, the bias and RMSE calculations were made based on the remaining number of successful trials. Table 1 shows the number of failures versus angular separation for the two-signal scenario. From this table, we can see that all three methods have some failures when the angular separation between emitters is 5 degrees. The Apodization and Chebyshev modifications have 7 failures out of 500 trials at a 5 degree angular separation between emitters and no failures as angular separation between emitters is increased. The two-step method has 15 failures in 500 trials at a 5 degree angular separation between emitters and has 8 failures in 500 trials at a 10 degree angular separation. Based solely on the number of failures, we can say that the Apodization and Chebyshev window modifications have an initial advantage when compared with the two-step modification. When bias and RMSE are computed, these failed trials are first removed.

**Figure 6:**
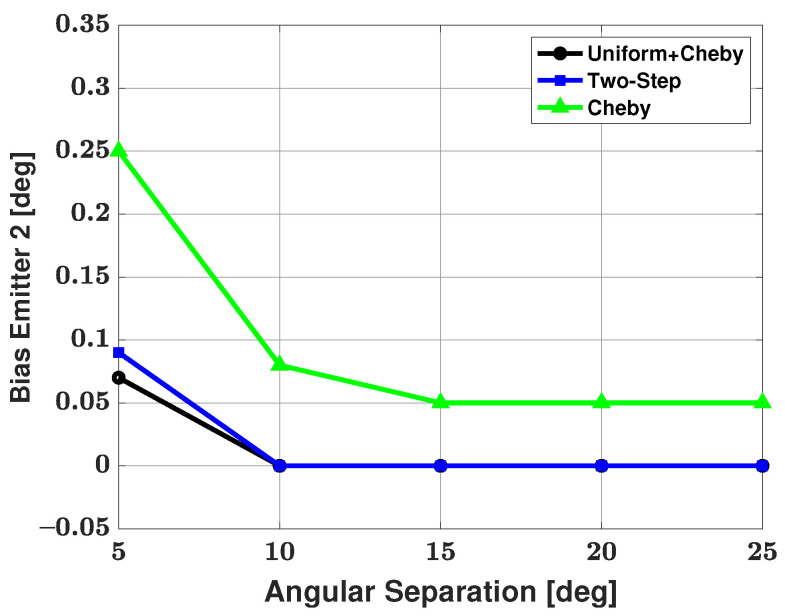
Bias in the estimated direction of the second, weaker emitter versus angular separation between the two emitters: the three proposed methods are used for estimation, SNR of emitter #1 = 25 dB and emitter #2 = 0 dB, respectively; 100 snapshots, and 500 trials.

**Figure 7:**
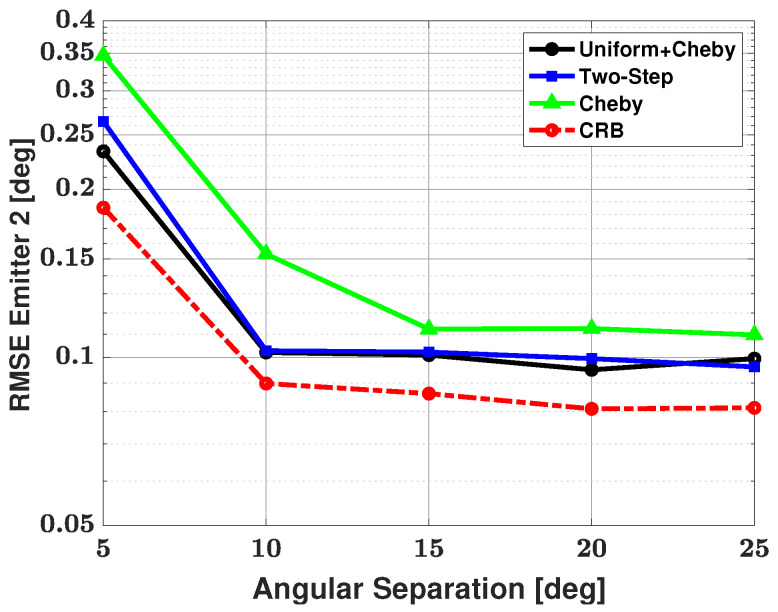
RMSE in the estimated direction of the second, weaker emitter versus angular separation between the two emitters: the three proposed methods are used for estimation, SNR of emitter #1 = 25 dB and emitter #2 = 0 dB, respectively; 100 snapshots, and 500 trials.

Figure 6 and Figure 7 show the bias and RMSE of the three modified methods, and the CRB for the weaker signal after failures have been removed. As expected, the strong signal has an RMSE that approaches the CRB for each method and is therefore not shown here. From the figures, we can see that the Apodization and two-step modifications have similar performances in terms of bias and RMSE and that the Chebyshev windowed modification has a larger bias and RMSE than the other two modifications. Based on the number of failed trials, we saw an advantage when using the Apodization and Chebyshev modifications rather than using the two-step modification. Based on bias and RMSE, we saw an advantage when using the Apodization and two-step modifications rather than the Chebyshev modification. Based on the number of failures, bias, and RMSE, one can note that the Apodization modification proved to be the most advantageous of all three modifications. The Apodization modification has the best overall performance of the three modifications, with small bias, RMSE, and number of failures.

### 5.2. Three-Signal Scenario

Next, we evaluated the performance of all three modifications with a more complex three-signal scenario. Signal #1 is incident to the antenna array at 25° and has an SNR of 18 dB. Signal #2 is incident to the antenna array at 60° and has an SNR of 25 dB. Signal #3 varied from 65° to 85°, with a step size of 5°, and has an SNR of 0 dB. One hundred snapshots were used in the DOA estimation. The three modified approaches are once again compared with each other and the CRB, with the weaker signal being the focus.

**Figure 8:**
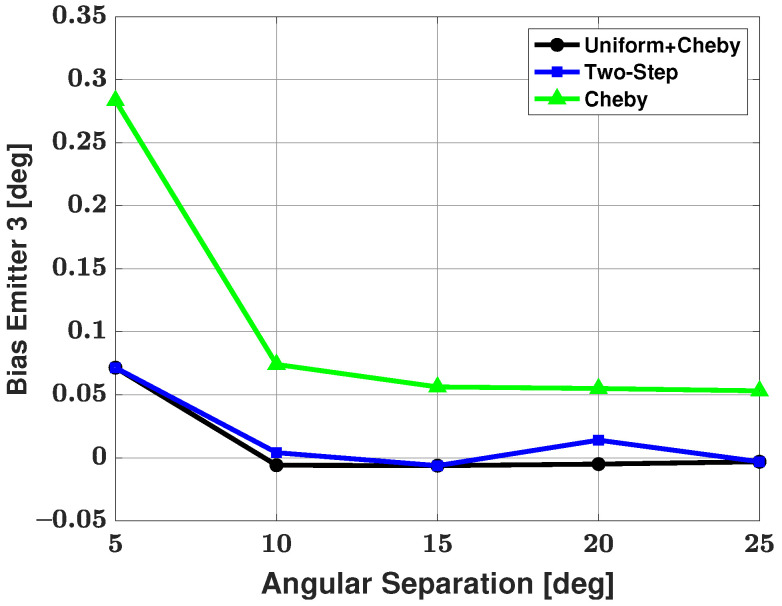
Bias in the estimated direction of the third, weakest emitter versus angular separation between emitters #2 and #3: the three proposed methods are used for estimation; SNR of emitter #1 = 18 dB, emitter #2 = 25 dB, and emitter #3 = 0 dB, respectively; 100 snapshots; and 500 trials.

**Figure 9:**
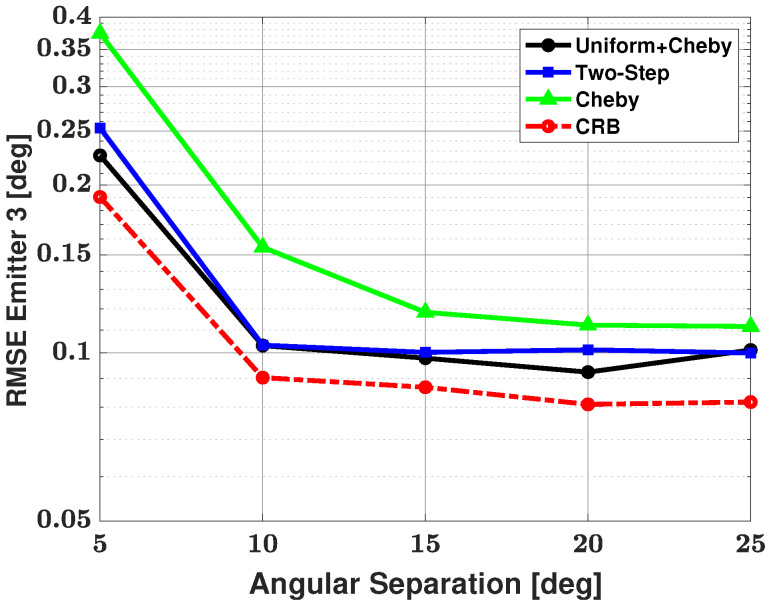
RMSE in the estimated direction of the third, weakest emitter versus angular separation between emitters #2 and #3: the three proposed methods are used for estimation; SNR of emitter #1 = 18 dB, emitter #2 = 25 dB, and emitter #3 = 0 dB, respectively; 100 snapshots; and 500 trials.

Table 2 shows the number of failures versus angular separation for the three-signal scenario. From the table, we can see that the Apodization and Chebyshev modifications both have 9 failures in 500 trials at a 5 degree angular separation between emitters and then no failures as angular separation is increased. The two-step modification has 22 failures in 500 trials at a 5 degree angular separation, 9 failures in 500 trials at a 10 degree angular separation, and no failures as angular separation is increased further. Based on this failure table, we can say that using the Apodization and Chebyshev modifications has a clear advantage in terms of number of failures over the two-step method. Bias and RMSE of all three modified methods and the CRB for the weaker signal after the failures have been removed are shown in Figure 8 and Figure 9, respectively. From these plots, one can note that the Apodization and two-step modifications have very similar bias and RMSE, with the Apodization modification having a slight advantage over the two-step modification. The Bias and RMSE of the Chebyshev windowed modification is significantly larger than that of the other two modifications. Again, one can look at the failure table and at the bias and RMSE plots and note that the Apodization modification has the best overall performance in terms of small number of failures, bias, and RMSE.

### 5.3. Four-Signal Scenario

Next, a fourth signal was added to the previous scene. Signal #1 is incident to the antenna array at 25° and has an SNR of 18 dB. Signal #2 is incident to the antenna array at 40° and has an SNR of 5 dB. Signal #3 is incident to the antenna array at 60° and has an SNR of 25 dB. Signal #4 varied from 65° to 85°, with a step size of 5°, and has an SNR of 0 dB. One hundred snapshots were used in the DOA estimation. This scenario now has two strong signals and two weaker signals. As there are two weaker signals, the bias and RMSE of both of those signals are shown.

**Figure 10:**
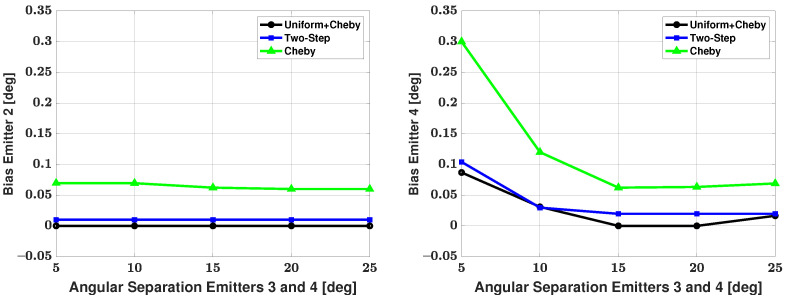
Bias in the estimated direction versus angular separation between emitters #3 and #4: emitter #2 (**left**) and emitter #4 (**right**); the three proposed methods are used for estimation; SNR of emitter #1 = 18 dB, emitter #2 = 5 dB, emitter #3 = 25 dB, and emitter #4 = 0 dB, respectively; 100 snapshots; and 500 trials.

**Figure 11:**
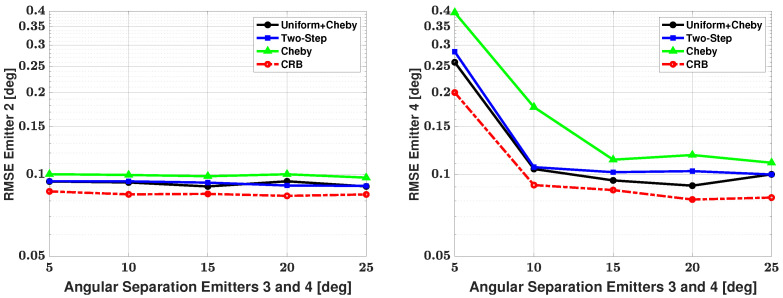
RMSE in the estimated direction versus angular separation between emitters #3 and #4: emitter #2 (**left**) and emitter #4 (**right**); the three proposed methods are used for estimation; SNR of emitter #1 = 18 dB, emitter #2 = 5 dB, emitter #3 = 25 dB, and emitter #4 = 0 dB, respectively; 100 snapshots; and 500 trials.

Table 3 shows the number of failures versus angular separation for the four-signal scenario. As there are now two weaker signals, failure to detect either of these weaker signals resulted in a failed trial. From the table, we can see that at a 5 degree angular separation between emitters, the Apodization and Chebyshev modifications have 9 failures in 500 trials and no additional failures as angular separation is increased. The two-step modification has 24 failures in 500 trials at a 5 degree angular separation, and the number of failures decreases until there are no failures after a 15 degree angular separation. One can note that this is the first time the two-step modification has had failures after a 10 degree angular separation. Based solely on the number of failures, we can say that the Apodization and Chebyshev modifications have an advantage when compared with the two-step modification. As there are now two weak signals, Figure 10 and Figure 11 show the bias and RMSE of all three modified methods and the CRB for signal #2 and signal #4 after the failures have been removed. From the plots, one can note that the Apodization and two-step modifications have similar bias and RMSE performances, with the Apodization modification having a slight advantage. Additionally, the plots show that the Chebyshev modification has significantly larger bias and RMSE when compared with the other two modifications. When considering bias, RMSE, and number of failures, we can say the Apodization modification again has the best overall performance of the three methods with small number of failures, bias, and RMSE.

## 6. Conclusions

In this paper, we presented a modification of the SDSR method that utilizes Apodization to successfully estimate DOA in the presence of signals with large dynamic ranges. The SDSR method was initially presented in an earlier paper [34]. We compared the performance of the Apodization modification with two other potential modifications to the SDSR method as well as with the CRB. One of those two additional modifications is the two-step SDSR method, where strong signals are identified and removed in the first step and weak signals are identified in the second step. The other SDSR modification is based on obtaining the Bartlett spectra using a low sidelobe window function. We showed that, in terms of number of failures, bias, and RMSE, the Apodization modification provided the best overall performance of the three modifications in the two-, three-, and four-signal scenarios.

## Figures and Tables

**Table 1:** Number of failures in 500 trials with respect to angular separation for a two-signal scenario. The SNR of signal #1 = 25 dB, the SNR of signal #2 = 0 dB, 100 snapshots, and 500 trials.

Angular Sep. [Deg]	Two-Step Approach	Uniform+Cheby	Cheby
5	15	7	7
10	8	0	0
15	0	0	0
20	0	0	0
25	0	0	0

**Table 2:** Number of failures in 500 trials with respect to angular separation for the three-signal scenario. The SNR of signal #1 = 18 dB, the SNR of signal #2 = 25 dB, the SNR of signal #3 = 0 dB, 100 snapshots, and 500 trials.

Angular Sep. [deg]	Two-Step Approach	Uniform+Cheby	Cheby
5	22	9	9
10	9	0	0
15	0	0	0
20	0	0	0
25	0	0	0

**Table 3:** Number of failures in 500 trials with respect to angular separation for the four-signal scenario. The SNR of signal 1 = 18 dB, the SNR of signal 2 = 5 dB, the SNR of signal 3 = 25 dB, the SNR of Signal 4 = 0 dB, 100 snapshots, and 500 trials.

Angular Sep. [deg]	Two-Step Approach	Uniform+Cheby	Cheby
5	24	9	9
10	13	0	0
15	8	0	0
20	0	0	0
25	0	0	0

## Data Availability

Data sharing not applicable. All data generated in this paper was done through simulation and can be recreated by following the methodology presented and using the parameters we have provided.
